# Schizophrenia and attendance in primary healthcare: a population-based matched cohort study

**DOI:** 10.1080/02813432.2019.1639927

**Published:** 2019-07-12

**Authors:** H. C. B. Nørgaard, H. Schou Pedersen, M. Fenger-Grøn, M. Vestergaard, M. Nordentoft, T. M. Laursen, O. Mors

**Affiliations:** a Psychosis Research Unit, Aarhus University Hospital, Risskov, Denmark;; b Department of Clinical Medicine, Aarhus University, Aarhus N, Denmark;; c Research Unit for General Practice & Section for General Medical Practice, Department of Public Health, Aarhus University, Aarhus C, Denmark;; d Psychiatric Center Copenhagen, University of Copenhagen, Hellerup, Denmark;; e National Centre for Register-based Research, Aarhus BSS, Aarhus University, Aarhus C, Denmark

**Keywords:** schizophrenia, primary health care, multimorbidity, mortality

## Abstract

**Objective:** Schizophrenia is associated with high mortality, somatic comorbidity and reduced life expectancy. The general practitioner (GP) plays a key role in the treatment of mental and physical multimorbidity. Nevertheless, it is unclear how much individuals with schizophrenia use primary healthcare. This study aims to investigate the yearly numbers of consultations in general practice for individuals with schizophrenia.

**Design and Setting:** We performed a population-based matched cohort study of 21,757 individuals with schizophrenia and 435,140 age- and gender-matched references from Danish National Registers. Monthly general practice consultations were analysed using a generalized linear model with log link and assuming negative binomial distribution.

**Main outcome measures:** Consultation rates in general practice up to17 years after index diagnosis.

**Results:** Individuals with schizophrenia attended their GP more than references throughout the study period. The cases had 82% (95% CI: 78-87) and 76% (95% CI: 71-80) more consultations in primary care after 1 year and 5 years, respectively. Individuals with both schizophrenia and comorbid somatic illness attended even more.

**Conclusion:** Individuals with schizophrenia are in regular contact with their GP, especially if they have comorbid illnesses. Whether an average of six consultations per year for individuals with schizophrenia is sufficient is up for debate. The study demonstrates a potential for an increased prevention and treatment of individuals with schizophrenia in general practice.

**KEY POINTS**
Schizophrenia is associated with high mortality, somatic comorbidity and reduced life expectancy. Little is known about the attendance pattern in primary care for individuals with schizophrenia.
•We found high attendance rates in primary care for individuals diagnosed with schizophrenia from index diagnosis and at least 17 years after diagnosis, which suggests opportunities for earlier intervention to improve their somatic health.
•We found an association between high illness comorbidity and increased risk of not attending the general practitioner. The most severely somatically and mentally ill individuals may thus be difficult to reach and support in the current healthcare system.

## Introduction

Individuals with schizophrenia have a 15–20 years reduced life expectancy compared to the general population [1–3]. Schizophrenia is strongly associated with a multifactorial higher risk of death [4–6]. Schizophrenia is characterized by both negative symptoms [loss of initiative, neglect of self-care] and psychotic symptoms, which often reduce social interaction. These characteristics often lead to a sedentary lifestyle, unhealthy diet and little exercising [7,8] and hence also increased risk of developing lifestyle-associated diseases. Furthermore, negative and psychotic symptoms can be barriers to seeking help and receiving the correct medical treatment [9,10]. Less frequent participation in screening [11] and undertreatment of somatic illness [12] also contribute to the high mortality. General practitioners [GPs] play a crucial role for individuals with multimorbidity as GPs are trained in managing several concurrent medical conditions [13]. Danish GPs handle a broad spectrum of illnesses and serve as gatekeepers to secondary care [12]. Still, individuals with schizophrenia are repeatedly hospitalised for preventable conditions [14]. It remains unclear why individuals with schizophrenia are underdiagnosed and poorly treated for somatic illness. The primary healthcare system may play a role, but little is known about the use of general practice.

We aimed to describe the primary healthcare utilisation in Danish general practice for individuals with incident schizophrenia and to evaluate whether certain groups of individuals are at higher risk of missing out in general practice.

## Materials and methods

### Design

We conducted a population-based matched comparative cohort study based on Danish nationwide registry data. The Danish Civil Registration System (CRS) [15] includes data for all Danish citizens on name, gender, date of birth, place of birth, citizenship and identity of parents. All records are continuously updated with information on vital status, place of residence and cohabitation status. This information can be linked through the civil registration number (CRN), which is assigned to all Danish citizens.

### Study population

We identified cases in the Psychiatric Central Research Register ([PCRR). We included all individuals aged 15–90 years diagnosed with schizophrenia in Denmark from January 1998 to June 2014. For each case with schizophrenia (ICD8 code 295 and ICD10 code F20) we randomly sampled twenty age- and gender-matched (± one month) individuals from the background population as references. Prevalent cases from 1969 (the year PCRR was established) until December 1997 were censored to ensure that only incident cases with a first-time diagnosis of schizophrenia were included. Information on gender and age was obtained from the CRS and information on migration was provided by Statistics Denmark. Sampling was done without replacement for each schizophrenia case but with replacement between schizophrenia cases, i.e. each person could serve as a reference for more than one schizophrenia case but only once for the same schizophrenia case. Also, reference persons could later be included as a schizophrenia case if they were given the diagnosis.

### Index date

For individuals diagnosed as inpatients (i.e. admitted to a psychiatric hospital immediately before first diagnosis of schizophrenia), the index date was defined as the first date of discharge from hospital after the diagnosis of schizophrenia. For individuals diagnosed as outpatients, the index date was defined as the first visit in an outpatient clinic after the registration of the diagnosis.

### National registers

The PCRR holds information on all inpatient contacts from 1969 and outpatient contacts from 1995, including diagnosis, date of treatment initiation, type of referral, place of treatment, municipality of residence and mode of admission.

All services provided by Danish GPs are registered prospectively in the Danish National Health Service Registry (NHSR). This register holds information on all contacts to general practice since 1990 [16]. Danish GPs have approximately 1600 listed patients each, including 4–7 individuals with schizophrenia. The registrations in the NHSR are considered to be very complete, as Danish GPs are paid on a tax-funded fee-for-service basis, which requires each GP to register all consultations to get reimbursed [16].

Information on all contacts to somatic hospital outpatient clinics for both cases and references was obtained from the Danish National Patient Register (NPR); which holds information on somatic diagnoses from 1977 and all somatic outpatient contacts from 1995 [17].

### Socioeconomic status

Information on marital/partner status and education status was obtained from Statistics Denmark and Danish education registers [18,19]. Marital/partner status was indexed as living with a partner (married, registered civil partnership or cohabiting) or living solitary.

The highest attained educational level was divided into three categories based on the International Standard Classification of Education (ISCED) developed by the United Nations: low (<10 years), medium (10–15 years) and high (>15 years) [20].

### Comorbidity

We obtained information on comorbidity using an earlier developed algorithm with 39 categorised somatic and mental disorders [21]. The disorders were identified by combining diagnosis codes (ICD10) with data on redeemed prescriptions for medication from the Register of Medicinal Product Statistics. We identified the number of comorbid illnesses (0, 1, or ≥2 comorbid illnesses) and furthermore determined whether cases and references had any of the four specified somatic illnesses: diabetes mellitus, hypertension, ischemic heart disease or lung disease (Tables 1–3). All baseline characteristics were collected from registers at the time of the index diagnosis and after five years.

**Table 1.** Cohort characteristics at baseline and five years after index date.

Measure	Baseline Schizophrenia (N)	Baseline References (N)	*p* Value	5 years Schizophrenia (N)	5 Years References (N)	*p* Value
Total	21,757	435,140		14,935*	311,550*	
Male	12,687 (58%)	253,470 (58%)		8621 (58%)	181,249 (58%)	
Female	9070 (42%)	181,400 (42%)		6314 (42%)	130,301 (42%)	
Education						
<10 years	13,834 (64%)	169,750 (39%)	<0.001	8969 (60%)	85,777 (28%)	<0.001
10–15 years	6415 (29%)	200,669 (46%)	<0.001	4834 (32%)	162,717 (52%)	<0.001
>15 years	1508 (7%)	64,721 (15%)	<0.001	1132 (8%)	63,056 (20%)	<0.001
Single/living alone	17,483 (81%)	215,221 (49%)	<0.001	11,820 (79%)	117,670 (38%)	<0.001
Married/ cohabitating	4265 (19%)	219,684 (51%)	<0.001	3071 (21%)	192,595 (62%)	<0.001
Comorbidity						
Number of diseases						
0	13,361 (62%)	356,556 (82%)		8480 (57%)	238,576 (77%)	
1	5557 (25%)	52,562 (12%)	<0.001	3715 (25%)	45,486 (15%)	<0.001
≥2	2839 (13%)	26,022 (6%)		2740 (18%)	27,488 (9%)	
Somatic diseases						
Diabetes mellitus	736 (3.4%)	8263 (1.9%)	<0.001	848 (5.7%)	8304 (2.7%)	<0.001
Hypertension	1221 (5.6%)	22,661 (5.2%)	<0.001	1315 (8.8%)	23,825 (7.6%)	<0.001
Ischemic heart disease	265 (1.2%)	3804 (0.90%)	<0.001	259 (1.7%)	4041 (1.3%)	<0.001
Lung diseases	620 (2.8%)	10,422 (2.4%)	<0.001	558 (3.7%)	8526 (2.7%)	<0.001

* Of the 21.757 cases at baseline 1023 (4,7%) persons had died five years after index date, and 5799 persons did not obtain five years observation time.

Of the 435.140 references at baseline 5190 persons had died (1,2%).

**Table 2.** Daytime consultations in General Practice during 1st and 5th year after index date.

Subgroups	1st year (Sz)	1st year (Ref)		5th year (Sz)	5th year (Ref)	
	Mean annual No Consultations	Mean annual No Consultations	1st yearIRR (95% CI)	Mean annual No Consultations	Mean annual No Consultations	5th yearIRR (95% CI)
All	5.84 (5.70;5.98)	3.28 (3.27;3.30)	1.82 (1.78;1.87)	6.05 (5.90;6.20)	3.55 (3.53;3.56)	1.76 (1.71;1.80)
Male	4.66 (4.50;4.82)	2.36 (2.34;2.37)	1.97 (1.90;2.04)	4.56 (4.39;4.73)	2.59 (2.57;2.61)	1.79 (1.73;1.86)
Female	7.47 (7.23;7.71)	4.58 (4.55;4.60)	1.64 (1.59;1.70)	8.08 (7.82;8.35)	4.88 (4.85;4.91)	1.71 (1.65;1.77)
Education						
<10 years (low)	6.15 (5.96;6.34)	3.63 (3.60;3.66)	1.75 (1.69;1.80)	6.31 (6.11;6.51)	4.03 (4.00;4.06)	1.64 (1.58;1.69)
10–15 years (medium)	5.26 (5.06;5.48)	3.09 (3.07;3.11)	1.69 (1.62;1.76)	5.59 (5.35;5.85)	3.30 (3.28;3.33)	1.68 (1.60;1.76)
>15 years (high)	5.46 (5.04;5.92)	2.98 (2.95;3.01)	1.79 (1.64;1.94)	5.78 (5.21;6.41)	3.10 (3.07;3.14)	1.85 (1.66;2.02)
Cohabitation						
Single/living alone	5.60 (5.44;5.76)	3.07 (3.05;3.09)	1.80 (1.75;1.85)	5.72 (5.56;5.89)	3.38 (3.36;3.41)	1.67 (1.62;1.72)
Married/cohabitating	6.79 (6.54;7.06)	3.49 (3.47;3.51)	1.89 (1.82;1.97)	7.34 (7.00;7.70)	3.69 (3.66;3.71)	1.93 (1.84;2.03)
Comorbidity						
Number of diseases						
0	4.62 (4.50;4.74)	2.77 (2.76;2.78)	1.68 (1.63;1.72)	5.19 (5.04;5.35)	3.06 (3.04;3.07)	1.69 (1.64;1.74)
1	6.31 (6.01;6.61)	4.61 (4.57;4.66)	1.46 (1.38;1.53)	6.41 (6.10;6.73)	4.99 (4.93;5.05)	1.41 (1.33;1.49)
≥2	10.70 (10.10;11.33)	7.70 (7.57;7.84)	1.47 (1.38;1.57)	10.19 (9.53;10.90)	7.97 (7.85;8.10)	1.39 (1.29;1.49)
Somatic Diseases						
Diabetes mellitus	11.69 (10.55;12.95)	7.37 (7.20;7.54)	1.65 (1.46;1.87)	10.97 (9.79;12.29)	7.44 (7.24;7.64)	1.50 (1.34;1.69)
Hypertension	10.55 (9.76;11.41)	6.76 (6.67;6.85)	1.67 (1.53;1.82)	9.98 (9.18;10.84)	7.05 (6.93;7.17)	1.54 (1.41;1.57)
Ischemic heart disease	10.35 (8.77;12.21)	7.10 (6.86;7.35)	1.52 (1.26;1.83)	11.35 (9.32;13.83)	7.27 (6.98;7.56)	1.64 (1.31;2.08)
Lung disease	11.78 (10.52;13.20)	5.85 (5.71;5.99)	2.03 (1.81;2.28)	11.16 (9.85;12.64)	5.86 (5.70;6.01)	1.93 (1.69;2.19)

IRR: incidence rate ratios; CI: confidence interval.

Sz: schizophrenia.

Ref: References.

**Table 3.** Relative Risk and Proportion of no GP consultations during 1st and 5th years after index date.

F2F contacts*	1st year after index date	Proportion year 1	Proportion year 1	5th year after index date	Proportion year 5	Proportion year 5
Baseline variables	RR (95% CI)	Reference	Schizophrenia	RR (95% CI)	Reference	Schizophrenia
All	0.68 (0.66;0.70)	24.18	20.77	0.79 (0.76;0.82)	24.57	21.17
Male	0.66 (0.64;0.69)	32.01	26.06	0.78 (0.75;0.81)	32.32	27.50
Female	0.74 (0.69;0.79)	13.22	13.41	0.82 (0.75;0.89)	13.79	12.51
Education						
<10 years (low)	0.67 (0.64;0.69)	22.94	19.72	0.80 (0.76;0.84)	22.85	20.52
10–15 years (medium)	0.74 (0.70;0.78)	24.62	21.99	0.84 (0.78;0.89)	25.10	21.95
>15 years (high)	0.81 (0.73;0.90)	26.03	25.13	0.82 (0.72;0.93)	27.24	23.44
Cohabitation						
Single/living alone	0.66 (0.64;0.69)	26.29	22.30	0.77 (0.74;0.81)	26.61	22.75
Married/cohabitating	0.58 (0.53;0.63)	22.06	14.46	0.66 (0.59;0.73)	22.82	14.87
Comorbidity						
Number of diseases						
0	0.73 (0.71;0.76)	26.91	23.29	0.81 (0.78;0.85)	26.98	22.98
1	0.95 (0.88;1.02)	14.25	19.34	1.18 (1.09;1.28)	15.17	20.35
≥2	1.12 (0.96;1.31)	6.71	11.64	1.36 (1.14;1.62)	8.45	12.57
Somatic diseases						
Diabetes mellitus	0.57 (0.39;0.83)	8.96	8.05	0.79 (0.51;1.22)	10.55	9.41
Hypertension	0.77 (0.59;1.01)	7.28	8.29	0.92 (0.68;1.24)	9.23	9.49
Ischemic heart disease	1.12 (0.71;1.76)	8.94	12.88	1.35 (0.80;2.27)	9.83	14.01
Lung disease	0.46 (0.40;0.84)	11.30	8.10	0.66 (0.45;0.97)	13.51	11.11

* Face-to face contacts; RR: relative risk; CI: confidence interval.

### Outcomes

Primary outcomes were consultations in general practice, daytime and out of hours. These were reported from index date and the following 17 years graphically and in absolute numbers one year and five years after the index date and described with personal characteristics.

Secondary outcomes were number of different services provided in general practice, e.g. blood sample tests, rapid strep tests, and, spirometer tests. Furthermore, we identified all patients with no GP consultations in the first and fifth year of observation. Finally, we examined the proportion of non-attenders in two settings: GP visit and GP visit plus somatic outpatient contact in secondary health care.

### Statistical methods

Individuals contributed with risk time from the index date and until the date of death or the end of the study period (June 2014), whichever came first. During follow-up, individuals were censored if hospitalized (somatic or psychiatric); they re-entered the study after hospital discharge. Individuals who emigrated were censored and re-entered the study after immigration to Denmark.

The number of consultations was analysed using a generalized linear model with a log link and assuming negative binomial distributed counts. These models yielded incidence rate ratios (IRRs). All analyses were adjusted for age, sex and calendar time. To account for heterogeneity between individuals in strata, we applied cluster robust variance estimation. The logarithm of risk time was used as offset. We analysed the number of face-to-face consultations (daytime, out-of-hours and both combined) and the number of diagnostic tests taken in different time periods. The follow-up period was divided into 1st month, 2nd month, 3rd month, 4th–5th month, 6th–11th month, 12th–17th month, 18th–23rd month, 3rd year, 4th year, 5th–6th year, 7th–12th year, and 13th–17th year after index date. The number of face-to-face consultations was also analysed for 1st and 5th observation year, and the consultations were stratified on pre-specified variables.

The dichotomous outcome of non-attendance was analysed using a binomial model with a log link. The probability${\text{ p}}_{\mathrm{ij}}\in \;\left[0;1\right]$ of non-attendance for individual i in period j was modelled as $p_{\mathrm{ij}}=\exp\left(X_{i}\times {B_{\mathrm{ij}}}^{T}*t_{\mathrm{ij}}\right),$ where ${\text{ t}}_{\mathrm{ij}}\in \;\left[0;1\right]$ denotes the risk time for individual i in period *j*, ${\text{ X}}_{i}$ denotes the matrix with 1 in the first column and the baseline values for the different variables used in the regression in the other columns, and $B_{\mathrm{ij}}$ denotes the corresponding coefficient matrix. Non-attendance was analysed for 1st and 5th observation year and was stratified on pre-specified variables. In a sensitivity analysis, we also included contacts to somatic outpatient clinics. Chi square tests were applied to test for differences in the baseline characteristics. Two-sided significance tests were performed for all analyses at 5% significance level.

## Results

The study included 21,757 individuals with schizophrenia (cases) and 435,140 age- and gender-matched references without schizophrenia (references). Cases and references had an average age of 34,6 years at index date.

**Figure 1.**
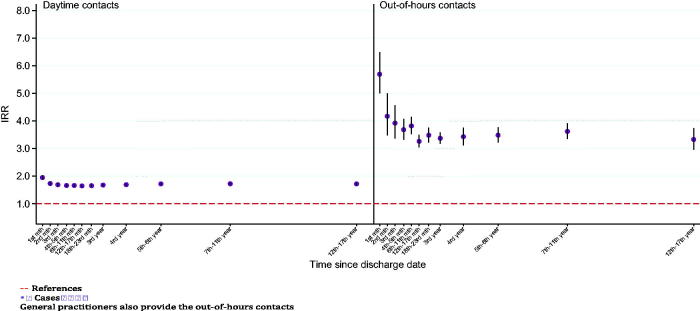
General practice daytime and Out-of-Hours consultations comparing patients with schizophrenia with the background population 1st and 5th years after index date.

Over all cases had more daytime and out-of-hours consultations in general practice than references (Figure 1), particularly during the first month after index date. During the first month cases had 95% more daytime consultations, IRR = 1.95 (95% CI: 1.88–2.03) this gradually decreased and settled at approximately 65% more consultations from four months after the index date and throughout the study period (up to 17 years). The numbers of out-of-hour GP services were 433% higher during the first month after the index date, IRR = 5.33 (95% CI: 4.69–6.05); then decreased and settled at approximately 300% more consultations for cases throughout the study period.

Cases had significantly more GP consultations than references during the first and the fifth observation year, regardless of gender, education, cohabitation and comorbidity. Cases had 82% (95% CI: 78–87) more consultations than references after one year and 76% (95% CI: 71–80) more after five years (6.05 vs. 3.55 annual consultations) (Table 2). After the first year, cases without any comorbidity had 68% more consultations in general practice, IRR = 1.68 (95% CI: 1.63–1.72), than references. This was significantly higher than the corresponding IRRs for cases and references with one comorbid illness, IRR = 1.46 (95% CI: 1.38–1.53), or two or more comorbid illness, IRR = 1.47(95% CI: 1.38–1.57).

At baseline and five years after index date, cases had a higher risk (38% and 43%) than references (18% and 25%) of having comorbid disorders (Table 1).

**Figure 2.**
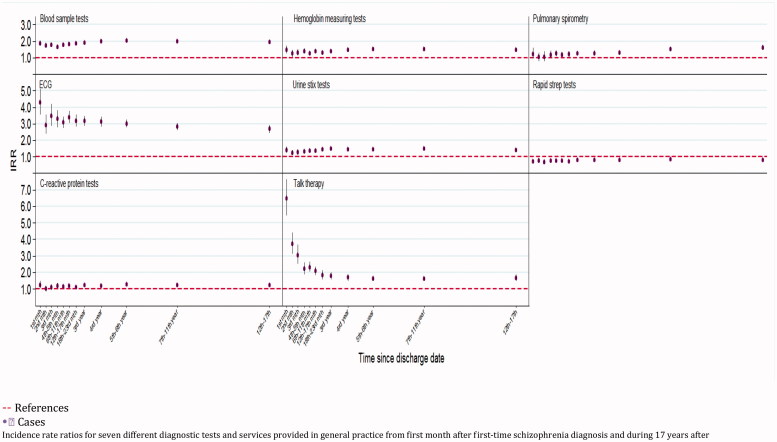
Services provided in general practice comparing patients with schizophrenia with the background population.

Cases had more consultations coded as; talk therapy, ECGs, blood tests and pulmonary tests. The number of performed haemoglobin level tests, urine stix tests and C-reactive protein tests were similar to the number for references, whereas fewer rapid strep tests were registered for cases (Figure 2).

Cases showed a lower probability of having no consultations to general practice after one year, relative risk (RR) = 0.68 (95% CI: 0.66–0.70), and five years, RR = 0.79 (95% CI: 0.76–0.82) (Table 3).

When stratified for; comorbid illnesses plus schizophrenia predicted a trend towards increasing likelihood of having no consultations to general practice. One year after the index date, the relative risk of having no consultations to general practice was 0.95 (95% CI: 0.88–1.02) for cases with one comorbid illness and 1.12 (95% CI: 0.96–1.31) for cases with for two or more comorbid illnesses (Table 3). Five years after the index diagnosis, the risk of having no consultations to general practice was statistically significantly higher for cases than for references (Table 3).

The sensitivity analysis showed a trend that cases with no consultations to general practice had a higher probability of having no somatic hospital outpatient contacts in addition to increasing comorbidity; five years after the index date, the cases had a significantly higher relative risk of having no consultations than the references (Data not shown).

## Discussion

### Principal findings

Cases had significantly more consultations to a general practitioner both during daytime and in out-of-hours consultations than references throughout the study period, indicating that general practice could play an important role in the identification and treatment of mental and physical diseases for these patients. This suggests that GPs are important healthcare professionals to service this patient group. The attendance in out-of hour’s services was particularly high for cases. This might not be so surprising with sleep impairment and circadian rhythm disturbances being a part of the schizophrenia syndrome. As well as these patients having difficulties planning their time or managing scheduled appointments. Furthermore the majority of the total consultations were daytime consultations demonstrated by the more narrow confidence intervals (Figure 1).

The relative difference between cases and references tended to decrease with increasing number of comorbidities. This may indicate that individuals with schizophrenia generally receive insufficient care when they have several somatic conditions or that the GP tends to manage several conditions during the same visit.

Cases had lower educational level and greater risk of comorbid illness and of living alone than references at baseline. This could indicate poor health conditions already from an early stage of their illness and outlines the severe risk profile for these vulnerable individuals as low educational level and solitary living is individual risk factors for early death.

Individuals with schizophrenia are also having more consultations in general practice before the diagnosis of schizophrenia [22]. Nonetheless, an increasing number of post-diagnostic GP consultations is not surprising as nearly 40% of cases already at baseline had one or more co-existing comorbid illnesses that require attention in general practice (Table 1). We do not know the exact reasons for the increased frequency of GP consultations. The cases had more face-to-face contact during daytime and out-of-hours care, but they also received more talk therapy, ECGs, blood tests, haemoglobin level measurements and pulmonary tests in general practice (Figure 2). The GPs thus managed many healthcare tasks, but for individuals with high comorbidity and a severe mental illness, it may be debatable whether the observed number of six yearly consultations (Table 2) is sufficient.

For both cases and references, the proportion with “no show” decreased with increasing numbers of comorbid illnesses. Yet, this decrease was less marked among cases, which left a trend of a higher risk of “no show” in general practice for cases with comorbid illness than references with comparable comorbidity both one year (11.64% versus 6.71% for persons with two or more comorbid illnesses) and five years after the index diagnosis (Table 3). Still, the cases censored due to temporary hospitalisation were included in these results. The relative risk of 1.12 (95% CI: 0.96–1.31) found in our models (Table 3) is thus likely to be a more accurate estimate. Our results show a trend, but these are not statistically significant one year after the index date.

The most somatically vulnerable group of individuals with schizophrenia appear to have greater risk of not attending their GP. This is a finding of concern as it implies a lower likelihood of receiving proper care for existing comorbid illnesses. We found an increased likelihood of “no show” for both consultations at the GP and at somatic hospital outpatient clinics with increasing number of comorbid illnesses; these figures were significant after five years (Data not shown). One explanation could be that individuals with schizophrenia have limited resources to prioritise consultations to healthcare professionals. The reduced visit rate in general practice for a small proportion of the cases with several comorbid illnesses may thus strengthen our hypothesis that somatic comorbid illnesses are undertreated, and this could be one of the contributors to the premature death seen in these individuals.

### Existing evidence

To our knowledge, this is the most comprehensive study to investigate attendance patterns in general practice for individuals with schizophrenia. Hetlevik et al. found that most individuals diagnosed with schizophrenia had regular contact with their GP, and patients with diabetes, obstructive pulmonary disease, or cardiovascular disease had more GP consultations than controls [23]. Our findings are comparable for regular GP contact, but our data showed that comorbid illness in cases with schizophrenia lowered the IRR of more GP consultations compared with references, which could also increase the risk of not attending the GP.

Osborn et al. found that individuals with severe mental illness (SMI) in British primary care were less likely to be screened for cardiovascular disease than individuals without SMI [24]. Smith et al. showed that individuals with schizophrenia have higher probability of having somatic comorbid illness and lower probability of having cardiovascular disease recorded in their GP medical record; this also suggests undertreatment of somatic disease [25]. A similar pattern was found in a study of 540 primary care practices in London [26]. We actually found a higher likelihood of ECG recording and blood tests taken in cases than in references; this could indicate a certain level of cardiovascular screening in Danish primary care or at least an awareness of the potential need for somatic screening.

### Strengths and limitations

Our study has several strengths. Selection bias is unlikely as we collected our data from Danish national registries, and the cohort was followed virtually without loss to follow-up. The data on consultations in general practice were collected prospectively and independently of the memory of patients, family members and GPs, which eliminated the risk of recall bias. The data from the NHSR are considered reliable, as all Danish GPs must report all provided services in this register to be reimbursed.

The study also has some limitations. The registers do not hold information on consultation content or the reason for making an appointment with the GP.

In the comparison of the consultation frequencies in individuals with schizophrenia and the gender-, age- and calendar time-matched reference population, it must be kept in mind that these two groups differ in other health-related aspects than the schizophrenia diagnosis. For this reason, the raw frequency counts were supplemented by regression analyses in which the two groups were compared under adjustment for gender, educational status, marital status and somatic comorbidities. Nevertheless, other unmeasured confounders may have affected the help-seeking pattern. The data did not allow us to control for potential residual confounders, e.g. severity of symptoms, relationship with the GP (who may be affected by earlier forced hospitalizations) and lifestyle (tobacco dependency, drug or alcohol abuse). This could have affected our results.

As our findings are based on nationwide data from a country with free and open access to general practice, they are generalizable to similar countries.

### Clinical implications

Most persons living in Denmark with a schizophrenia diagnosis attend their GP regularly after the initial diagnosis and continue to attend the GP, but undertreatment of somatic illness is not solved with this alone.

Our results leave several questions unanswered: Should individuals with schizophrenia have even more GP consultations to ensure proper treatment of their mental and somatic illnesses? Should they gain more from the consultations? Schizophrenia is a risk factor for more hospitalisations that probably could have been prevented by proper care in primary care, which supports the need for additional consultations or greater benefit from GP consultations.

Our findings confirm that individuals with schizophrenia remain in continuous attendance in general practice from the prodromal phase and several years after the first diagnosis. As nearly 40% have at least one comorbid illness at the time of their index diagnosis of schizophrenia, the GP has a big responsibility and many opportunities to improve the somatic health in these individuals.
